# On Saponin as a Local Anæsthetic

**Published:** 1874-04

**Authors:** 


### ARTICLE VIII.

### *On Saponin as a Local Anæsthetic.*

The *London Medical Record* quotes from Dr. Kohler on this subject:—

Saponin is obtained from many plants of natural orders. Silenæ (*e.g.* Saponaria of ficinalis) the Polygalaceæ (*e.g.* Polygala senega) of the Spireæ (Rosaceæ) (*e.g.* Quillaja saponaria) and the Sapotaceæ (Cortex monesiæ); it is an amorphous white powder, with neutral reaction and sweetish taste; it is soluble in water, forming a foaming fluid like soap-suds. The experiments made on it proved its local effects as well as those on the muscles and nervs of the extremities by subcutaneous injections, on the exposed hearts of frogs, on the intestines, and on the nervous centres by direct application to these organs; then the general effects from injections into the jugular veins; and lastly, the symptems produced by its introduction into the stomach.

The most important local effects are as follows. Five minutes after the application of a few drops of a concentrated solution, there occur perfect suspension of the reflex irritability of the part selected, and paralyses of both motor and sensory nerve-filaments

Shortly afterwards, the muscles of the part lose their power responding to chemical, mechanical, or electric irritants; and this may occur somewhat independently of the nerves. The nerve-trunks, and afterwards the nervous centres, do not become effected till larger quantites of the solution are applied, and then probably by absorption, as the effects become general. The capillaries, at the spot selected for injection, become greatly contracted, and so do the larger vessels, such as the vena cava and aorta, when the saponin is applied directly to them. When it is applied directly to the heart, the beats of this organ gradually become less frequent, and then cease altogether. This effect does not depend upon irritation of the terminal branches of the vagi, but on paralysis of the accelerator nerves (sympathetic), which thus raises the 'tonus' of the vagus filaments. Finally paralysis of the cardiac ganglia themselves (*i.e.* of those imbedded in the muscles of the heart) ensues. In a like manner, direct application of the solution to the abdominal organs first paralyzes their muscles and then their nerves. Local application to the nervous centres induces, at last, complete paralysis of these organs. This spreads peripheraly from the spinal cord; and, continuing to affect the medulla oblongata and the cerebrum, produces asphyxia (stoppage of respiration), deep coma, and dilatation of the pupils. The series of experiments made by injecting saponin into the vena cava of warm-blooded animals produced these effects; diminution of blood-pressure, succeeding a slight temporary increase of it; reduction of temperature and of the frequency of both respirations and cardiac contractions. The effects on respiration and upon blood pressure are produced through the nervous centre for these functions. The spsnal cord and the peripheral muscles and nerves are not paralyzed by these injections into the veins. Lastly, when saponin is introduced into the stomach, blood pressure is reduced, and pulse, respiration and temperature all sink, through slowly; paralyses of the extremities (as in the injections into the veins also) does not occur in this case. No alteration in either the quaniitiy or quality of the excreta have been observed. Clinical practice only can decide weather saponin may play a great part in surgery as a local anæsthetic.—*Med. and Surg. Reporter.*

